# Consumption of Plant Foods and Its Association with Cardiovascular Disease Risk Profile in South Africans at High-Risk of Type 2 Diabetes Mellitus

**DOI:** 10.3390/ijerph192013264

**Published:** 2022-10-14

**Authors:** Tatum Lopes, Annalise E. Zemlin, Jillian Hill, Zandile J. Mchiza, Nasheeta Peer, Rajiv T. Erasmus, Andre P. Kengne

**Affiliations:** 1 Non-Communicable Diseases Research Unit, South African Medical Research Council, Cape Town 7505, South Africa; 2 Division of Chemical Pathology, Department of Pathology, Faculty of Medicine and Health Sciences, University of Stellenbosch, Cape Town 7505, South Africa; 3 National Health Laboratory Service (NHLS), Tygerberg Hospital, Cape Town 7505, South Africa; 4 Department of Medicine, University of Cape Town, Cape Town 7925, South Africa

**Keywords:** plant foods, cardiovascular disease risk factors, type 2 diabetes mellitus

## Abstract

We assessed the distribution and association of cardiovascular disease (CVD) risk factors by plant foods consumption in individuals at high-risk for type 2 diabetes mellitus. This cross-sectional study utilized baseline data of 693 participants in the South African Diabetes Prevention Programme. Participants underwent a physical examination, biochemical analysis, and dietary assessment using a single non-quantified 24-h recall. Group comparisons were conducted to explore the distribution and associations of common CVD risk factors by plant foods consumption. The mean age of the participants was 51 years, with 81% being females. Consumers of yellow-coloured vitamin A-rich vegetables and tubers and maize had significantly lower systolic blood pressure, fasting insulin, low-density lipoprotein cholesterol, triglycerides, and fibrinogen levels. Cereals consumption increased the likelihood of obesity (OR = 1.72 95% CI [1.09, 2.70] *p* = 0.019) while the consumption of white roots and tubers decreased the likelihood of obesity (AOR = 0.64 95% CI [0.41, 1.00] *p* = 0.048). This study reported the consumption of some healthy plant foods with lower levels of, and decreased risk for, some CVD risk factors. A further in-depth investigation is needed to understand these associations.

## 1. Introduction

The consumption of plant foods is beneficial because it supplies the body with high-quality micronutrients, macronutrients, dietary fibre, and bioactive compounds that function as antioxidants to protect against non-communicable diseases (NCDs) such as cardiovascular disease (CVD) and type 2 diabetes mellitus (T2DM), among other conditions [1,2]. The beneficial plant foods include fruits and vegetables, which are the most extensively studied [1,3], whole grains, legumes, and nuts. Moreover, the current literature advocates that the type of plant foods consumed dictates the benefits obtained [4], because there are healthy and unhealthy plant foods, for example, refined grains and sugar-sweetened beverages. 

Studies on plant foods have been conducted in the high-income countries (HICs) of Europe and the United States with little research emanating from Africa and other low-and-middle income countries (LMICs). Most nutrition-related studies in Africa [5] have focused on assessing nutrition adequacy during early childhood and nutrient deficiencies associated with food insecurity in African populations [6,7]. There is a lack of sufficient research on the patterns of plant foods consumed in Africa and other LMICs, and very little is known about its association with CVDs and their cardiometabolic risk factors [8]. Such research is important because of changing dietary patterns, with the greater availability and intake of energy-dense foods high in saturated and trans fats, sugar, and salt; these changes in diets are attributable to urbanization and globalization, etc. [9,10].

The uptake of unhealthy dietary patterns contributes to increasing obesity levels and subsequent T2DM and CVDs. Recent literature has highlighted the rising burden of CVDs and their risk factors in African populations [11]. Compared with HICs, a higher proportion of premature mortality attributable to CVDs is occurring in LMICs, such as in Africa where these deaths are mainly observed in younger adults between the ages of 30 and 69 years [11,12,13]. The latter is concerning because it affects the working-age population in Africa who are likely breadwinners and thereby exacerbates poverty [11]. 

The importance of diets as a contributor to health has been proven, e.g., some plant foods have been found to lessen the burden of NCDs such as T2DM [1,2,14,15]. Therefore, this study, conducted in South Africans at high-risk for T2DM, examined the (1) distribution of plant food consumption by socio-demographic and behavioural factors and (2) associations of plant food consumption with hypertension, T2DM, dyslipidaemia, obesity, and subclinical inflammation.

## 2. Materials and Methods

### 2.1. Study Design and Population

The South African Diabetes Prevention Programme (SA-DPP) was conducted between August 2017 and July 2019 in participants without known T2DM residing in Cape Town, in the Western Cape province of South Africa. This community-based study, conducted in 16 low socioeconomic areas, comprised an equal number of mainly Black African and Mixed-Ancestry communities [16]. 

Participants at high risk for T2DM were identified on the African diabetes risk score and recruited into the study [17]. High risk was determined by age, the presence of hypertension, and abdominal obesity. Trained fieldworkers administered a brief questionnaire and conducted blood pressure (BP) and anthropometric assessments to evaluate participants’ risk of developing T2DM. Those identified as being at high risk for T2DM were invited to attend a dedicated research clinic at the South African Medical Research Council (SAMRC), Cape Town. 

Study participants were aged 25 to 65 years, Black African or Mixed-Ancestry, and fluent in at least one of the local languages i.e., English, Afrikaans, or isiXhosa. Individuals who were pregnant/breastfeeding, taking medications that could affect glucose tolerance, receiving tuberculosis treatment, bedridden, suffering from any severe mental illnesses or had substance abuse symptoms were excluded from the study.

Study participation was voluntary and written consent was obtained from those who agreed to participate in the study. Ethical approval was received from the relevant committees. The SA-DPP study was approved by the SAMRC Human Research Ethics Committee [EC018-7/2015]. The current study examined the baseline cross-sectional data and is part of a doctoral research project that sought ethical approval from the Stellenbosch University Health Research Ethics Committee [S19/03/056]. 

### 2.2. Clinic-Based Assessments

Study participants were asked to fast for at least 10 h before they arrived at the research clinic at the SAMRC for further evaluations that included: (1) an oral glucose tolerance test (OGTT) and other biochemical analyses, (2) physical examination, (3) and an administered questionnaire.

#### 2.2.1. Oral Glucose Tolerance Test and Biochemical Analysis

An OGTT was performed according to the WHO guidelines and fasting plasma and 2-h glucose levels were collected [18]. Glycated haemoglobin (HbA1c) analyses were performed on whole blood specimens by high-performance liquid chromatography (Biorad Variant Turbo, BioRad, South Africa). Fasting serum specimens were collected for the lipogram analysis: total cholesterol (TC), high-density lipoprotein cholesterol (HDL-C), and triglyceride (TG) levels were analyzed using the Roche Modular auto analyser and enzymatic colorimetric assays. Low-density lipoprotein cholesterol (LDL-C) was calculated using the Friedewald formula [19]: [LDL-C] = [TC] − [HDL-C] − [TG ÷ 5]. Fibrinogen analysis was performed on the Fully Automated Coagulation Analyzer, ACL Top (Instrumentation Laboratory, United States) using the plasma derived from the fasting citrate specimen. The blood specimens were analyzed at PathCare Reference Laboratory in Cape Town, South Africa which is an ISO 15189 accredited laboratory.

#### 2.2.2. Physical Examination

Standardized techniques were used to obtain the anthropometric data while participants were dressed in light clothing and without shoes. Weight measurements were taken using calibrated digital SECA scales with weights recorded to the nearest 0.1 kg (kg). Height was measured in centimetres (cm), using a fixed SECA stadiometer (2.2 m). Waist circumference (WC) was measured to the nearest 0.1 cm with the tape measure parallel to the floor and at the mid-point of the lower border of the last rib and upper border of the iliac crest. BP was measured on the left arm while the participants were resting and seated. Three readings were recorded at 2-min intervals using automated monitors with appropriate cuff sizes (Omron M6 Comfort, OMRON HEALTHCARE Co. Ltd., Kyoto, Japan) [16].

#### 2.2.3. Interview

The questionnaire was available in an electronic format (through a personal digital assistant [PDA]) and was administered by trained field workers in the participants’ preferred language. The following data were obtained: general information, socio-demographic information, and dietary history. 

##### Dietary Assessment

Dietary data were collected using a single non-quantified 24-h recall. The questionnaire was administered using a two-step approach. In step 1, the participants were asked to recall which food items they consumed during the previous 24 h “I want you to try and remember what you ate and drank yesterday from the moment you woke up in the morning, right through until you went to bed again last night?”. The reported food items were recorded according to the mealtimes of the day (breakfast, lunch, and supper). Subsequently, in step 2 those food items listed in step 1 were categorized according to food groups that were listed in a tabular format. As a follow-up question, the participants were asked “Besides the foods and drinks you consumed yesterday, did you have any of the following food and/or drink at any time during the day, yesterday?”. The table in step 2 consisted of 18 food groups with examples of the food items from each food group. Participants’ responses were coded and counted by assigning the following codes: 1 = yes or 0 = no for each of the food groups listed. 

The food groups were outlined as per the Food and Agriculture Organization of the United Nations (FAO) dietary assessment guide [20]. From the plant food groups, we included vitamin A-rich fruit, other fruit, dark green leafy vegetables, other vegetables, yellow-coloured vitamin A-rich vegetables and tubers, white roots, and tubers, cereals, beans, and peas, and nuts and seeds. Intake of sweets, spices, condiments, and beverages were not included in this analysis. The food consumption frequencies reported in this study only assessed the distribution of plant food groups that were defined as healthy (Table 1). Depending on the ethnicity of the study population in South Africa [21], maize meal porridge is a staple food [22]. Therefore, considering the high intake of maize in this sample, the grains food group was categorized into the cereals and maize food groups to better reflect the usual dietary intake of the study participants [23]. 

#### 2.2.4. Definitions and Calculations

Table 1 describes the 10 food groups that were assessed individually to determine the distribution of plant foods consumption. There is currently no standardized definition available for plant food consumption. In the current study plant foods consumption was based on a single recall of food items consumed the previous 24 h before the clinic visit. The latter may inconsistently rank individuals as consumers or non-consumers of plant foods because it is subject to day-to-day variation. The rationale for utilising a 24-h recall to assess plant foods consumption is based on the homogenous diet of the sampled population [23], having low purchasing power to consume a variety of food groups or drastically deviate from their day-to-day meals [24]. Therefore, based on evidence from previous studies in our setting, i.e., the assumption that the diet of our sampled population remains fairly stable will minimize inconsistently ranking individuals as plant food consumers. The average of the second and third systolic and diastolic BP measurements was used to determine the presence of hypertension. The 2020 International Society of Hypertension Global Hypertension Practice Guidelines was used to define hypertension as systolic BP ≥ 140 mmHg and/or diastolic BP ≥ 90 mmHg [25] and/or self-reported hypertension. WHO/IDF guidelines were used to define T2DM as fasting glucose ≥ 7.0 mml/L and/or 2-h plasma glucose value ≥ 11.1 mmol/L [26]. Dyslipidaemia was defined as LDL-C ≥ 3.0 mmol [27] and/or self-reported high blood cholesterol. Body mass index (BMI) was calculated as weight (kg)/[height × height] (m^2^). Normal weight was defined as a BMI between 18.5 and 24.9, overweight as a BMI from 25.0 to 29.9 and obesity as a BMI ≥ 30.0 kg/m^2^ according to the WHO classification [28]. Subclinical inflammation was defined using fibrinogen levels > 5 g/L [29,30].

### 2.3. Statistical Analysis

Continuous data are presented as means and standard deviations for normally distributed variables, medians and 25–75th percentiles for skewed variables or counts and percentages for categorical data. The Shapiro–Wilk and Kolmogorov–Smirnov tests were conducted to assess normality including visual checking of histogram plots. Group comparisons used the chi-square test and equivalents for qualitative variables, analysis of the variance (ANOVA) and non-parametric equivalents for continuous variables. Univariable and multivariable logistic regressions were conducted to derive the measures of association between CVD risk factors (hypertension, T2DM, dyslipidaemia, obesity and subclinical inflammation) and the consumption of plant foods (vitamin A-rich fruit, other fruit, dark green leafy vegetables, other vegetables, yellow-coloured vitamin A-rich vegetables and tubers, white roots and tubers, cereals, maize, legumes, nuts and seeds) while controlling for the effect of potential confounding factors such as age, sex and ethnicity (sociodemographic), and smoking and alcohol consumption (behavioural risk factors). The IBM SPSS Statistics version 27 software was used for the data analysis and *p*-values less than 0.05 were regarded as statistically significant. The precision to detect the prevalence of plant foods consumption was calculated using the following formula:

$$c=\sqrt{\frac{Z^{2\;}\times p\times \left(1-p\right)}{N}}$$

where *Z* is 1.96, *p* is the prevalence and N is the sample size.

## 3. Results

### 3.1. Characteristics of Study Population

Our initial sample consisted of 702 participants who were at high-risk for T2DM; after data cleaning, we excluded nine individuals with missing dietary intake data and/or health outcome variables. Our final sample of 693 participants included 131 males and 562 females with a mean age of 51 years. Table 2 outlines the characteristics of the study participants by sex and overall characteristics. Fifty-nine percent of participants self-reported that they were Black African and 41% were Mixed-Ancestry. Study participants had a reasonably fair level of education, with 75% attending a secondary/tertiary learning institution; education level did not differ significantly by sex (*p* = 0.791). Nearly half of the participants were unemployed (46%), and 72% had a household income of less than R3200 a month (approx. $218 at an exchange rate of 14.622) with a significantly higher prevalence of females reporting that they were unemployed and earned less than males. The prevalence of tobacco smoking, and alcohol consumption were significantly higher in males compared with females: tobacco smoking: 35% vs. 23%, *p* = 0.003 and alcohol use: 70% vs. 50% *p* < 0.001.

### 3.2. Distribution of Plant Foods Consumption

**Figure 1:**
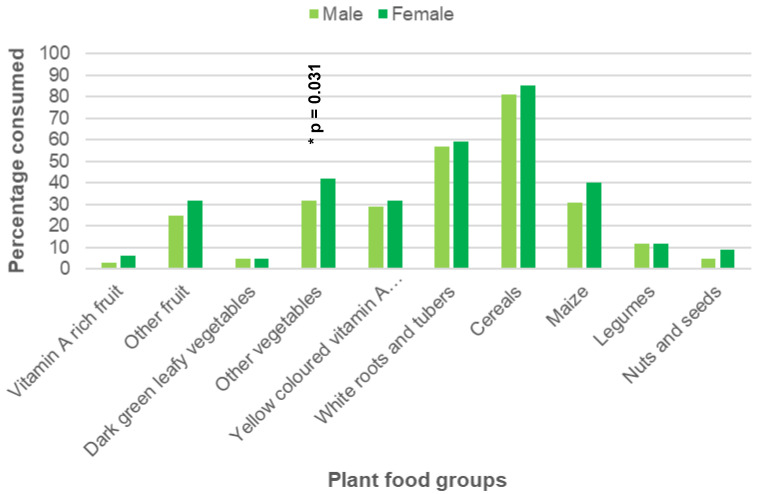
The distribution of plant foods consumption for males and females. The asterisk (*) indicates that the *p*-value was statistically significant (*p* < 0.05).

Table 2 and Figure 1 depict the distribution of the plant food groups consumed on the day of recall. The most frequently consumed were cereals (84%), followed by white roots and tubers (59%), other vegetables (40%) and maize (39%). The only difference in intake between males and females was the higher consumption of other vegetables in females (*p* = 0.031). 

### 3.3. Distribution of CVD Risk Factors by Plant Foods Consumption and Sex

Mean and median levels of CVD risk factors by plant food consumption are depicted in Table 3. Consumers vs. non-consumers of yellow-coloured vitamin A-rich vegetables and tubers, which are micronutrient dense, had lower systolic BP (125 vs. 128 mmHg, *p* = 0.030) and TG (1.2 vs. 1.3 mmol/L, *p* = 0.028) levels. 

Consumers compared with non-consumers of maize, a fibre and micronutrient dense food, had lower levels of LDL-C (3.0 vs. 3.2 mmol/L, *p* = 0.011), TG (1.2 vs. 1.3 mmol/L, *p* = 0.023) and fibrinogen (3.6 vs. 3.8 g/L, *p* < 0.001). Maize consumers also had lower fasting insulin levels compared with non-consumers (7.8 vs. 9.6 mIU/L, *p* < 0.001). 

Conversely, the consumers of other vegetables had higher 2-h glucose (7.2 vs. 6.7 mmol/L, *p* = 0.041) and HbA1c (6.1 vs. 6.0 %, *p* = 0.046) than non-consumers. There was no significant difference in the distributions of BMI and WC by plant food consumption. None of the CVD risk factors was significantly different between consumers and non-consumers of the following plant foods: white roots and tubers, cereals, dark green leafy vegetables, other vegetables, legumes, and nuts and seeds.

There were no significant differences in the distribution of CVD risk factors by plant foods consumption except for a single finding. Consumers of cereals had a higher prevalence of obesity (86% vs. 14%, *p* = 0.018) (Table S1, Supplementary Material).

The prevalence of hypertension was high at 63% with higher rates in females (65%) compared with males (54%), *p* = 0.027 (Table 4). Sixty-one percent of participants had dyslipidaemia with a similar prevalence by sex. Seventy-seven percent of the participants were obese, and a higher prevalence was observed in females (*p* < 0.001). 

### 3.4. Association of CVD Risk Factors by Plant Foods Consumption

The binary logistic regression analyses shown in Table 5, report the unadjusted odds ratios (ORs) and adjusted odds ratios (AORs) with 95% CI. In the unadjusted models, cereals consumption was positively associated with obesity (OR: 1.72, 95% CI 1.09–2.70; *p* = 0.019), however, the association became non-significant after controlling for confounders. In the adjusted models, white roots and tubers consumers had a 36% lower likelihood of being obese (AOR: 0.64, 95% CI 0.41–1.00; *p* = 0.048). Upon further adjustment for all healthy plant foods consumed, there was, however, no change in the magnitude or direction of the associations between cereals and/or white roots and tubers consumption as predictors of obesity. None of the plant food groups were significantly associated with the other CVD risk factors of hypertension, T2DM, dyslipidaemia or subclinical inflammation. 

## 4. Discussion

To our knowledge, this is the first community-based study to focus on the distribution and associations of healthy plant foods consumption with CVD risk factors in South African adults at high-risk for T2DM. This study demonstrated that micronutrient- and fibre-dense plant foods were beneficial; consumers of yellow-coloured vitamin A-rich vegetables and tubers had lower systolic BP and TG levels, while consumers of maize had lower fasting insulin, LDL-C, TG, and fibrinogen levels. Consumption of white roots and tubers was associated with a lower likelihood of being obese. Of note was that the consumption of cereals was associated with a more than 1.5-fold increased risk of obesity. 

These findings are in keeping with the literature, mainly from HICs, which highlights the differential impact of healthy versus unhealthy plant foods on cardiometabolic health [4]. The beneficial effects of healthy plant foods are derived from quality macronutrients (unrefined carbohydrates, protein, and unsaturated fat), and are high in micronutrients (vitamins and minerals), fibre, and antioxidants such as carotenoids. These lower BP, and improve lipid profiles, glycaemic control, and weight management, and reduce the risk of CVD mortality. Conversely, unhealthy plant foods are usually nutrient-poor carbohydrates, low in vitamins and fibre, high in saturated fat, salt and/or sugar, thus increasing CVD risk factors and the incidence of CVDs [1,4]. 

The lower prevalence of TG in consumers of yellow-coloured vitamin A-rich vegetables and tubers is in keeping with reviews by Mirmiran et al. [31] and Marcelino et al. [32] that reported reductions in TG with the intake of yellow-coloured vegetables. The latter contain antioxidants such as carotenoids (β-carotene) that aid in controlling lipid metabolism and adipocyte production [31,32,33]. Our findings add to the current literature on plant food-based diets because none of the studies in the abovementioned reviews was conducted in Africa. Most research in Africa on diets has considered animal food sources of vitamin A, such as liver and eggs, and perhaps neglected the more traditional, readily available, and affordable plant food sources of vitamin A such as yellow-coloured vitamin A-rich vegetables and tubers [34]. The current study highlights that vitamin A can be obtained from plant foods by consuming yellow-coloured vitamin A-rich vegetables and tubers in financially constrained South Africans. These findings are in keeping with a local study which recommended that vitamin A-rich plant foods be incorporated as a staple food in South Africa to improve nutritional status [35]. Considering that only about a third of participants reported consuming yellow-coloured vitamin A-rich vegetables and tubers the previous day, initiatives may be needed to educate consumers and encourage the intake of such healthy plant foods. 

The better lipid profiles with lower LDL-C and TG levels, and lower fasting insulin and fibrinogen levels among maize consumers are in keeping with studies conducted in HICS such as the United States [36,37]. Although none of the latter studies that reported on the health benefits of maize was conducted in African populations, these studies highlighted that the high-amylose content of maize, which makes it fibre-dense, and is characteristic of South African maize, likely contributes to the beneficial effects [38]. 

That grains such as cereals consumption were a strong predictor of obesity in this study may be because of the intake of commercial cereals which are usually highly refined, and high in trans-fat, added sugar and salt; this promotes the development of obesity [39,40]. These findings contrast with the literature on HICs and may be related to differences in the quality of grains and cereals consumed [41,42]. Further research is needed to understand these differences.

This study found that consumers of white roots and tubers were protected against obesity and accords with the literature from the United States and the United Kingdom [43,44,45]. However, this contrasts with the literature in general, which may be related to cooking practices e.g., the intake of fried vs. baked or boiled foods with the consumption of fried foods contributing to obesity [46]. Food preparation methods were not assessed in this study and need to be examined in future studies in this high-risk population. 

The dietary patterns described in this study accord with studies conducted in this population over a decade ago, demonstrating that healthy plant foods consumption is still part of local diets [9,10]. The earlier studies reported that cereals/roots/tubers were the primary foods consumed in the Western Cape [23], with females generally consuming more plant foods than males [47]. 

One of the major strengths of this study was the large sample size (n = 693). In addition to this, the current study fills a gap in the literature by adding to the limited knowledge on the association between plant foods consumption and CVD risk factors in South Africa among other LMICs.

A limitation of this study was the use of single versus multiple non-quantified 24-h dietary recalls to assess plant foods consumption. A single 24-h recall is prone to recall bias and does not account for day-to-day variations in food intake. Utilizing dietary data that is non-quantified is a further limitation because it does not reflect the amount of food consumed per food group. Moreover, bearing in mind that the majority of our study participants consumed cereals on the day of recall and that this is a high-risk aging cohort susceptible to disease, our outcomes may have been influenced by other confounders that were not adjusted for. Possible confounders include medication use, energy intake, and/or their overall dietary patterns which may have included the consumption of animal food groups. Therefore, the dietary correlations that were significantly associated with the measured CVD risk factors in our study population should be interpreted with caution and within the context of our study aims, which focused on healthy plant foods. In addition to this, the current study cannot explain or account for any food interactions that occurred when the study participants consumed mixed meals (i.e., plant and animal foods) during the days preceding, following or on the day of recall. Due to the cross-sectional nature of this study, we cannot establish causal associations between plant food dietary intake and CVD risk factors. Furthermore, our findings do not apply to the general population but are relevant and/or comparable to studies that have investigated similar exposure and outcome measures i.e., plant foods consumption in relation to CVD risk factors amongst adults with a predisposition for T2DM amongst other NCDs [1,48,49]. 

Based on our cross-sectional findings and the abovementioned limitations, we recommend that future studies utilize quantified dietary measurement tools, administered on more than one occasion with a longer recall period, if feasible, to lower the risk of bias and account for the overall diet rather than single food groups. Also important, is to determine the quantities of plant foods consumed, the cooking methods employed and their associations with CVD risk factors. Furthermore, longitudinal studies are needed to understand the contribution of plant foods consumption to the incidence of CVD risk factors in South Africa and other African and LMICs. 

## 5. Conclusions 

The current study showed that the consumption of micronutrient and fibre-dense plant foods was beneficial in South Africans at high-risk for T2DM. The latter foods included yellow-coloured vitamin A-rich vegetables and tubers, and maize which was associated with significantly lower levels of some CVD risk factors such as systolic BP, fasting insulin, LDL-C, TG, and fibrinogen. Furthermore, we found that white roots and tubers consumption was associated with a lower risk of obesity, while the consumption of cereals (e.g., refined grains) was associated with a greater risk of obesity. Further research is needed to understand these associations. 

## Figures and Tables

**Table 1:** List of healthy plant food groups and description of local food items.

Plant Food Groups		Food Items	Descriptions of Local Food Items
FRUITS AND VEGETABLES	Vitamin A-rich fruit	mango, pawpaw, yellow peach	
	Other fruit	apple, apricot, banana, grapes, grapefruit, guava, lemon, lime, naartjie, orange, peach, pear, plum, pineapple, prickly pear, raspberries, strawberries, watermelon, wild fruit	Naartjie: mandarin orange or tangerine
	Dark green leafy vegetables	spinach, marog, imifino	Marog/imifino: green leafy vegetable usually Amaranthus species; also referred to as African spinach
	Other vegetables	beetroot, broccoli, cabbage, cauliflower, cucumber, green beans, green peas, green pepper, lettuce, mushrooms, onions, tomato	
	Yellow-coloured vitamin A-rich vegetables and tubers	butternut, carrot, pumpkin, dark-orange sweet potato	
	White roots and tubers	potato, sweet potatoes, potato (either cooked, mashed, fried, potato salad, fries/slap chips )	Slap chips: potato, French fried
GRAINS	Cereals	sorghum, rice, pasta, oats, Mabela, Morvite, wheat, bread, home-made bread, breakfast cereals	Mabela: a sorghum meal, dry porridge Morvite: an instant sorghum porridge, instant
	Maize	maize-meal porridge (stiff, crumbly, or soft), fermented maize porridge, samp, mealie rice, whole maize (corn-on-the cob)	Samp: corn, dried, yellow maize; also similar to Corn grits/maize grits
	Legumes	dried beans, sugar beans, baked beans, lentils, dried peas, cowpeas, split peas	
	Nuts and seeds	peanuts, nuts, sunflower seeds, pumpkin seeds	

**Table 2:** Characteristics of study population.

Characteristics	Precision	Overall	Sex Male (n = 131) Female (n = 562)		*p*-Value
**Age, mean years (SD)**		51 (8.95)	53	51	0.429
**Ethnicity, n (%)**					
Black African		410 (59)	76 (58)	334 (59)	
Mixed-Ancestry		283 (41)	55 (42)	228 (41)	0.773
**Level of education, n (%)**					
None/Primary (Grade 1–7)		170 (25)	33 (26)	137 (25)	
Secondary/Tertiary		519 (75)	96 (74)	423 (75)	0.791
**Employment status, n (%)**					
Employed		171 (29)	42 (39)	129 (27)	
Self-employed		59 (10)	19 (18)	40 (8)	
Part-time employed		59 (10)	8 (7)	51 (11)	<0.001 *
Unemployed		271 (46)	39 (36)	232 (48)	
Other		26 (4)	0 (0)	26 (5)	
**Income status, n (%)**					
R0–R3200		494 (72)	85 (65)	409 (73)	
R3201–R6400		117 (17)	22 (17)	95 (17)	0.043 *
R6401–R51,200		79 (11)	23 (18)	56 (10)	
**Current smoker, n (%)**		174 (25)	46 (35)	128 (23)	0.003 *
**Alcohol consumption, n (%)**		373 (54)	92 (70)	281 (50)	<0.001 *
**Plant food groups consumed on the day of recall, n (%)**					
Vitamin A-rich fruit	0.018	38 (6)	4 (3)	34 (6)	0.175
Other fruit	0.034	210 (30)	33 (25)	177 (32)	0.157
Dark green leafy vegetables	0.016	36 (5)	7 (5)	29 (5)	0.932
Other vegetables	0.036	280 (40)	42 (32)	238 (42)	0.031 *
Yellow-coloured vitamin A-rich vegetables and tubers	0.035	218 (32)	38 (29)	180 (32)	0.503
White roots and tubers	0.037	406 (59)	74 (57)	332 (59)	0.588
Cereals	0.086	582 (84)	106 (81)	476 (85)	0.288
Maize	0.034	268 (39)	41 (31)	227 (40)	0.054
Legumes	0.024	84 (12)	16 (12)	68 (12)	0.971
Nuts and seeds	0.020	55 (8)	7 (5)	48 (9)	0.223

SD: Standard deviation. Data are presented as counts (n) and percentages (%). *p*-values with an asterisk (*) are statistically significant, level of significance set at <0.05. Other: Homemaker or student. Vitamin A-rich fruit: mango, pawpaw, yellow peach. Other fruit: apple, apricot, banana, grapes, grapefruit, guava, lemon, lime, naartjie, orange, peach, pear, plum, pineapple, prickly pear, raspberries, strawberries, watermelon, wild fruit. Dark green leafy vegetables: spinach, marog, imifino. Other vegetables: beetroot, broccoli, cabbage, cauliflower, cucumber, green beans, green peas, green pepper, lettuce, mushrooms, onions, tomato. Yellow-coloured vitamin A-rich vegetables and tubers: butternut, carrot, pumpkin, dark-orange sweet potato. White roots and tubers: potato, sweet potatoes, potato (cooked, mashed, fried, potato salad, fries/slap chips). Cereals: sorghum, rice, pasta, oats, mabela, morvite, wheat, bread, home-made bread, breakfast cereals. Maize: maize-meal porridge (stiff, crumbly, or soft), fermented maize porridge, samp, mealie rice, whole maize (corn-on-the cob). Legumes: dried beans, sugar beans, baked beans, lentils, dried peas, cowpeas, split peas. Nuts and seeds: peanuts, nuts, sunflower seeds, pumpkin seeds.

**Table 3:** Mean and median level of CVD risk factors in consumers and non-consumers of plant food groups.

Parameters	Laboratory Ref. Intervals	Maize			Yellow-Coloured Vitamin A-rich Vegetables and Tubers			Other Vegetables			Other Fruit		
		No	Yes	*p*-Value	No	Yes	*p*-Value	No	Yes	*p*-Value	No	Yes	*p*-Value
**Age in years,**		51	51		51	52		51	51		51	53	
**mean (SD)**		(8.7)	(9.4)	0.663	(8.9)	(9.1)	0.257	(8.9)	(9.0)	0.992	(9.2)	(8.4)	0.013 *
**HYPERTENSION**													
SBP in mmHg,		128	126		128	125		128	126		126	129	
mean (SD)		(18.6)	(19.2)	0.418	(19.4)	(17.4)	0.030 *	(19.8)	(17.4)	0.127	(18.2)	(20.3)	0.161
DBP in mmHg,		85	84		85	84		85	84		85	85	
mean (SD)		(11.6)	(11.0)	0.081	(11.6)	(10.7)	0.141	(11.5)	(11.2)	0.145	(11.5)	(11.0)	0.653
**T2DM**													
Fasting glucose in mmol/L, mean (SD),		5.4	5.4		5.4	5.3		5.3	5.5		5.3	5.5	
	3.5–5.5	(1.4)	(1.8)	0.947	(1.5)	(1.6)	0.759	(1.2)	(2.0)	0.059	(1.5)	(1.7)	0.076
2-h glucose in mmol/L, mean (SD),		7.0	7.0		6.9	6.8		6.7	7.2		6.7	7.2	
		(3.2)	(3.6)	0.517	(3.3)	(3.4)	0.615	(2.9)	(3.9)	0.041 *	(3.2)	(3.7)	0.067
HbA1c in %,		6.0	6.0		6.0	6.0		6.0	6.1		6.0	6.1	
mean (SD)	3.9–6.1	(0.9)	(1.1)	0.811	(0.9)	(1.1)	0.679	(0.7)	(1.2)	0.046 *	(0.9)	(1.1)	0.156
Fasting insulin in mIU/L,		9.6	7.8		9.0	8.5		8.7	8.8		8.7	8.7	
median [25th;75th percentile]	2.1–10.4	[6.4; 13.9]	[4.8; 11.6]	<0.001 *	[5.8; 13.2]	[5.5; 12.5]	0.214	[5.7; 12.5]	[5.7; 13.6]	0.346	[5.7; 13.0]	[5.7; 13.0]	0.956
**DYSLIPIDAEMIA**													
TC in mmol/L,		5.1	5.0		5.1	5.1		5.1	5.0		5.0	5.1	
mean (SD)	<5.0	(1.2)	(1.2)	0.064	(1.2)	(1.2)	0.511	(1.2)	(1.2)	0.764	(1.2)	(1.2)	0.703
HDL-C in mmol/L,		1.3	1.3		1.3	1.3		1.3	1.3		1.3	1.3	
mean (SD)	>1.2	(0.3)	(0.4)	0.078	(0.3)	(0.4)	0.339	(0.3)	(0.3)	0.858	(0.3)	(0.3)	0.800
LDL-C in mmol/L,		3.2	3.0		3.2	3.1		3.2	3.1		3.1	3.2	
mean (SD),	<3.0	(1.0)	(1.0)	0.011 *	(1.0)	(1.0)	0.454	(1.0)	(1.0)	0.699	(1.0)	(1.0)	0.512
TG in mmol/L,		1.3	1.2		1.3	1.2		1.2	1.3		1.2	1.3	
median [25th;75th percentile]	<1.7	[1.0; 1.8]	[0.9; 1.6]	0.023 *	[0.9; 1.7]	[0.9; 1.5]	0.028 *	[0.9; 1.7]	[0.9; 1.7]	0.492	[0.9; 1.7]	[1.0; 1.7]	0.284
**OBESITY**													
BMI in kg/m^2^,		36	36		36	36		36	36		36	35	
mean (SD)		(7.1)	(7.7)	0.625	(7.4)	(7.3)	0.925	(7.7)	(6.9)	0.737	(7.5)	(7.0)	0.132
WC in cm,		105	103		105	103		104	104		105	103	
mean (SD)		(11.7)	(13.2)	0.099	(12.4)	(12.2)	0.207	(12.7)	(11.8)	0.779	(12.4)	(12.1)	0.184
**SUBCLINICAL**													
**INFLAMMATION**													
Fibrinogen in g/L,		3.8	3.6		3.7	3.7		3.7	3.7		3.7	3.7	
mean (SD)	2.2–5.0	(0.7)	(0.7)	<0.001 *	(0.7)	(0.6)	0.649	(0.7)	(0.6)	0.983	(0.7)	(0.6)	0.533

Data presented as means and (SD: standard deviation) or median and [25th; 75th percentile]. *p*-values with an asterisk (*) are statistically significant, level of significance set at <0.05. SBP: Systolic blood pressure, DBP: Diastolic blood pressure, HbA1c: Glycated haemoglobin, TC: Total cholesterol, HDL-C: High-density lipoprotein cholesterol, LDL-C: Low-density lipoprotein cholesterol, TG: Triglycerides, BMI: Body mass index, WC: Waist circumference. Maize: maize-meal porridge (stiff, crumbly, or soft), fermented maize porridge, samp, mealie rice, whole maize (corn-on-the cob). Yellow-coloured vitamin A-rich vegetables and tubers: butternut, carrot, pumpkin, dark-orange sweet potato. Other vegetables: beetroot, broccoli, cabbage, cauliflower, cucumber, green beans, green peas, green pepper, lettuce, mushrooms, onions, tomato. Other fruit: apple, apricot, banana, grapes, grapefruit, guava, lemon, lime, naartjie, orange, peach, pear, plum, pineapple, prickly pear, raspberries, strawberries, watermelon, wild fruit.

**Table 4:** Prevalence of CVD risk factors.

Characteristics	Overall	Sex Male (n = 131) Female (n = 562)		*p*-Value
**Hypertension, n (%)**	432 (63)	71 (54)	361 (65)	0.027 *
**T2DM, n (%)**	70 (10)	14 (11)	56 (10)	0.807
**Dyslipidaemia, n (%)**	415 (61)	78 (61)	337 (61)	0.902
**BMI categories, n (%)**				
Normal weight	29 (4)	21 (16)	8 (1)	
Overweight	129 (19)	62 (47)	67 (12)	<0.001 *
Obese	533 (77)	48 (37)	485 (87)	
**Subclinical inflammation, n (%)**				
Elevated fibrinogen	21 (3)	6 (5)	15 (3)	0.219

Data are presented as counts (n) and percentages (%). *p*-values with an asterisk (*) are statistically significant, level of significance set at <0.05. BP: Blood pressure. Hypertension: elevated systolic BP ≥ 140 mmHg and/or diastolic BP ≥ 90 mmHg and/or self-reported hypertension. T2DM: screen-detected on oral glucose tolerance tests with fasting glucose ≥ 7.0 mmol/L and/or 2-h glucose ≥ 11.1 mmol/L. LDL-C: Low-density lipoprotein cholesterol. Dyslipidaemia: elevated LDL-C ≥ 3.0 mmol/L and/or self-reported high cholesterol. BMI: Body mass index. Normal weight: BMI 18.5–24.9 kg/m^2^. Overweight: BMI 25.0–29.9 kg/m^2^. Obese: BMI ≥ 30 kg/m^2^. Elevated fibrinogen: >5 g/L.

**Table 5:** OR and AORs with 95% CIs for CVD risk factors across plant food groups.

Parameters	White Roots and Tubers				Cereals			
	OR	p-Value	AOR	p-value	OR	p-Value	AOR	p-Value
	(95% CIs)		(95% CIs)		(95% CIs)		(95% CIs)	
**Hypertension**	0.92 (0.67; 1.26)	0.586	0.91		0.70 (0.46; 1.06)	0.094	0.70	0.089 †
			(0.67; 1.26)	0.580 †			(0.46; 1.06)	
			0.89				0.69	0.082 ‡
			(0.65; 1.23)	0.490 ‡			(0.45; 1.05)	
**T2DM**	1.09 (0.66; 1.80)	0.749	1.05		1.16 (0.57; 2.34)	0.683	1.10	
			(0.63; 1.75)	0.848 †			(0.54; 2.23)	0.801 †
			1.08				1.11	
			(0.65; 1.79)	0.775 ‡			(0.55; 2.26)	0.769 ‡
**Dyslipidaemia**	1.00 (0.73; 1.35)	0.978	0.97		0.97 (0.64; 1.47)	0.874	0.96	
			(0.70; 1.34)	0.835 †			(0.62; 1.49)	0.860 †
			0.97				0.96	
			(0.70; 1.34)	0.853 ‡			(0.62; 1.49)	0.864 ‡
**Obesity**	0.77 (0.53; 1.11)	0.160	0.64		1.72 (1.09; 2.70)	0.019 *	1.56	
			(0.41; 1.00)	0.048 †,*			(0.90; 2.70)	0.114 †
			0.66				1.55	
			(0.42; 1.03)	0.070 ‡			(0.88; 2.71)	0.129 ‡
**Subclinical inflammation**	0.65 (0.27; 1.55)	0.332	0.65		0.57 (0.21; 1.60)	0.289	0.56	
			(0.27; 1.56)	0.339 †			(0.20; 1.60)	0.280 †
			0.66				0.57	
			(0.27; 1.58)	0.351 ‡			(0.20; 1.60)	0.285 ‡

† Model 1: adjusted for age, sex, and ethnicity. ‡ Model 2: adjusted for age, sex, and ethnicity, smoking and alcohol consumption. *p*-values with an asterisk (*) are statistically significant, the level of significance was set at <0.05. Hypertension: systolic blood pressure ≥ 140 mmHg and/or diastolic blood pressure ≥ 90 mmHg and/or self-reported hypertension. T2DM: fasting glucose ≥ 7.0 mml/L and/or 2-h plasma glucose value ≥ 11.1 mmol/L. LDL-C: Low-density lipoprotein cholesterol. Dyslipidaemia: LDL-C ≥ 3.0 mmol/L and/or self-reported high blood cholesterol. BMI: Body mass index. Obesity: BMI ≥ 30.0 kg/m^2^. Subclinical inflammation: fibrinogen levels > 5 g/L. White roots and tubers: potato, sweet potatoes, potato (cooked, mashed, fried, potato salad, fries/slap chips). Cereals: sorghum, rice, pasta, oats, mabela, morvite, wheat, bread, home-made bread, breakfast cereals.

## Data Availability

Data supporting reported results can be requested from A.P.K but will currently not be made available publicly as the SA-DPP study is still on-going.

## Supplementary Materials

The following supporting information can be downloaded at: https://www.mdpi.com/article/10.3390/ijerph192013264/s1, Table S1: Significant differences in the distribution of CVD risk factors by plant food groups.
