# Supplementary material for: Consumption of Plant Foods and Its Association with Cardiovascular Disease Risk Profile in South Africans at High-Risk of Type 2 Diabetes Mellitus
Source: Int J Environ Res Public Health. 2022 Oct 14;19(20):13264. doi: 10.3390/ijerph192013264 (PMC9603168; doi:10.3390/ijerph192013264)
Supplement: Supplementary file 1 [file ijerph-19-13264-s001.zip › ijerph-1924088-supplementary.pdf]

Table S1. Significant differences in the distribution of CVD risk factors by plant food groups.

**Parameters**	**Cereal consumption**		
	**No**<br>**N (%)**	**Yes**<br>**N (%)**	***p*-Value**
Hypertension (n=432)	77 (18)	355 (82)	0.108
T2DM (n=70)	10 (14)	60 (86)	0.683
Dyslipidaemia (n=415)	69 (17)	346 (83)	0.413
Obesity (n=533)	76 (14)	457 (86)	0.018*
Subclinical inflammation (n=21)	5 (24)	16 (76)	0.283

Data presented as counts (n) and percentages (%). p-values with an asterisk (*) are statistically significant, level of significance set at < 0.05. Hypertension: SBP ≥ 140 mmHg and/or DBP ≥ 90 mmHg and/or self-reported hypertension. T2DM: fasting glucose ≥ 7.0 mml/L and/or 2-hour plasma glucose value ≥ 11.1 mmol/L. Dyslipidaemia: LDL-C ≥ 3.0 mmol/L and/or self-reported high blood cholesterol. Obesity: BMI ≥ 30.0 kg/m^2. Subclinical inflammation: Fibrinogen levels > 5 g/L. Cereals: sorghum, rice, pasta, oats, mabela, morvite, wheat, bread, home-made bread, breakfast cereals.
